# Supplementary material for: Simultaneous macroscale and microscale wave–ion interaction in near-earth space plasmas
Source: Nat Commun. 2022 Sep 23;13:5593. doi: 10.1038/s41467-022-33298-6 (PMC9508155; doi:10.1038/s41467-022-33298-6)
Supplement: Supplementary file 1 — Supplementary Information [file 41467_2022_33298_MOESM1_ESM.pdf]

Supplementary Information for

Simultaneous Macroscale and Microscale Wave-ion Interaction in near-Earth Space Plasmas

Z.-Y. Liu, Q.-G. Zong*, R. Rankin, H. Zhang, Y. F. Wang, X.-Z. Zhou, S.-Y. Fu, C. Yue, X.-Y. Zhu, J. Pollock, S. A. Fuselier, G. Le
Correspondence to: qgzong@pku.edu.cn

This PDF file includes:

Supplementary Fig. 1 to 7
Supplementary Methods
Supplementary References

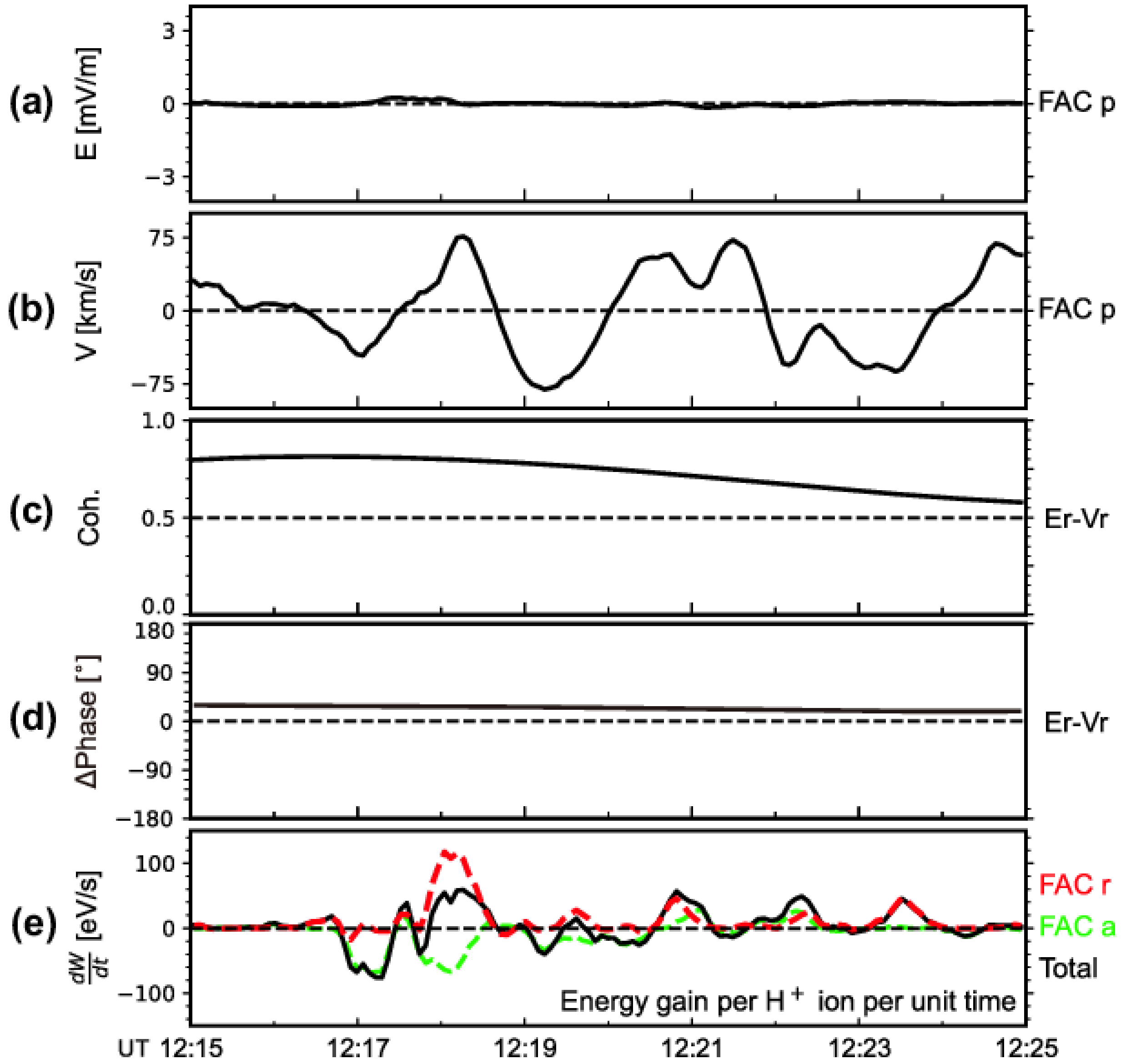

Supplementary Fig. 1. ULF wave-ion interactions in the September 5, 2015, event. Fast mode data is used here. (**a** and **b**) The parallel components of the ULF-wave electric field and the bulk velocity of 9.8-26.6 keV ions from FPI, respectively. (**c** and **d**) A cross-wavelet analysis of the ULF-wave radial electric field and the 9.8-26.6 keV ion radial bulk velocity, with panels c and d showing the correlation coefficient and phase difference at ~3.4 min (the period of the ULF waves), respectively. (**e**) H^+ ion energy gain from ULF waves per ion per unit time, averaged over the energy range of 9.8-26.6 keV. The black curve corresponds to the total energy gain, while the red and green curves represent the contribution from the radial and azimuthal components, respectively.

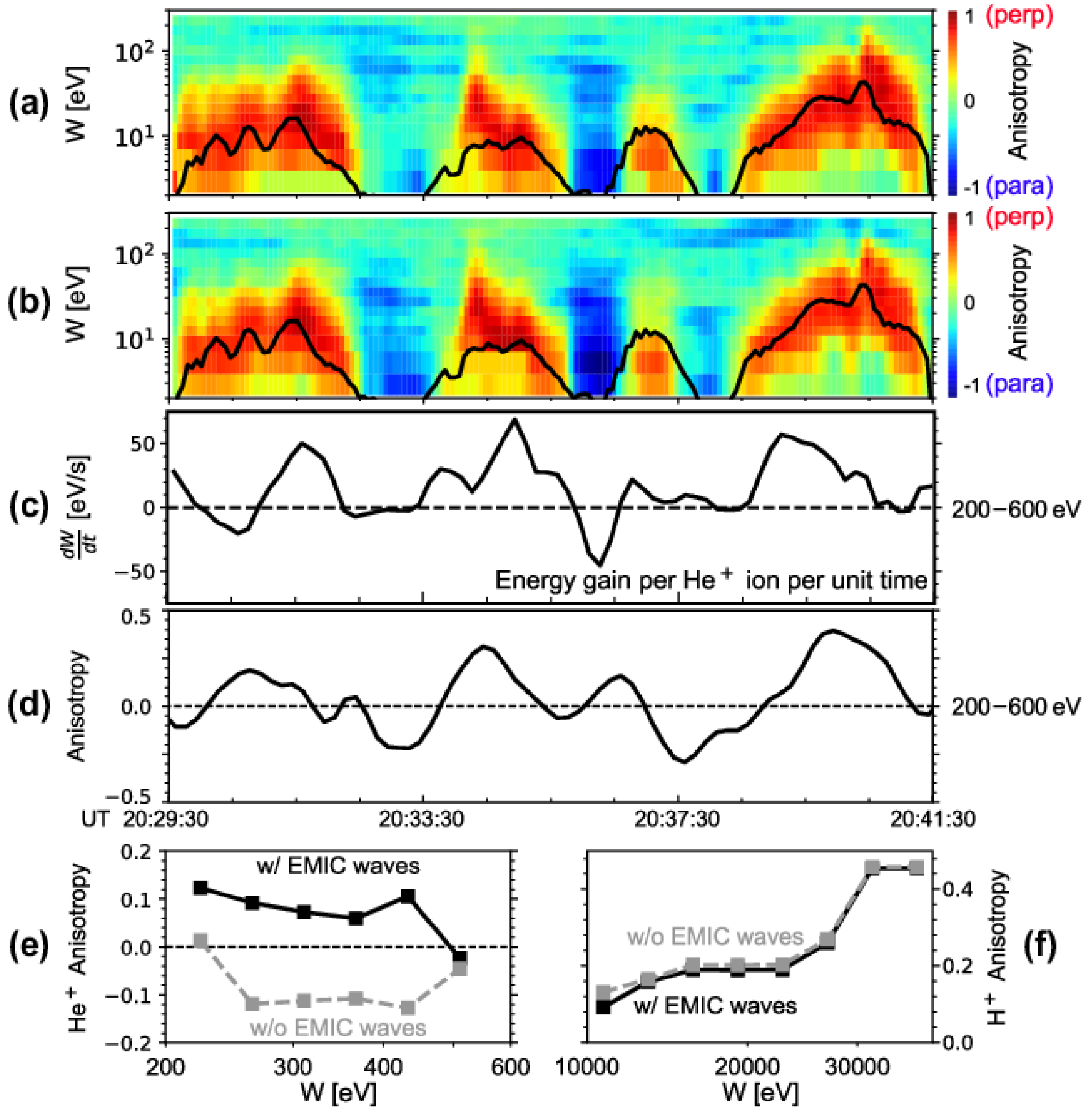

Supplementary Fig. 2. Ion in the January 7, 2019, event. FPI fast mode data is used in panels a and b. HPCA fast survey mode data is used in panels c-f. (**a** and **b**) Anisotropy of cold ions in the rest frame of the spacecraft, defined in the same manner as Fig. 1g but in the rest frame of the spacecraft. (**c**) Energy gain from ULF waves per ion per unit time for He^+ ions in the energy range of 200-600 eV. (**d**) He^+ ion anisotropy averaged over 200-600 eV in the rest frame of the plasma. (**e**) He^+ ion anisotropy in the rest frame of the plasma when EMIC waves are present (black) and absent (grey). The corresponding time intervals are marked by bar below Supplementary Fig. 3c. (**f**) H^+ ion anisotropy in the rest frame of the plasma, with the same format as panel e but for higher energy where EMIC wave-H^+ ion cyclotron resonance is possible (the minimum resonant energy here is ~9.0 keV). Since H^+ ion anisotropy does not change much with and without the presence of the EMIC waves, H^+ ions should contribute little to the growth of the EMIC waves, if there is any.

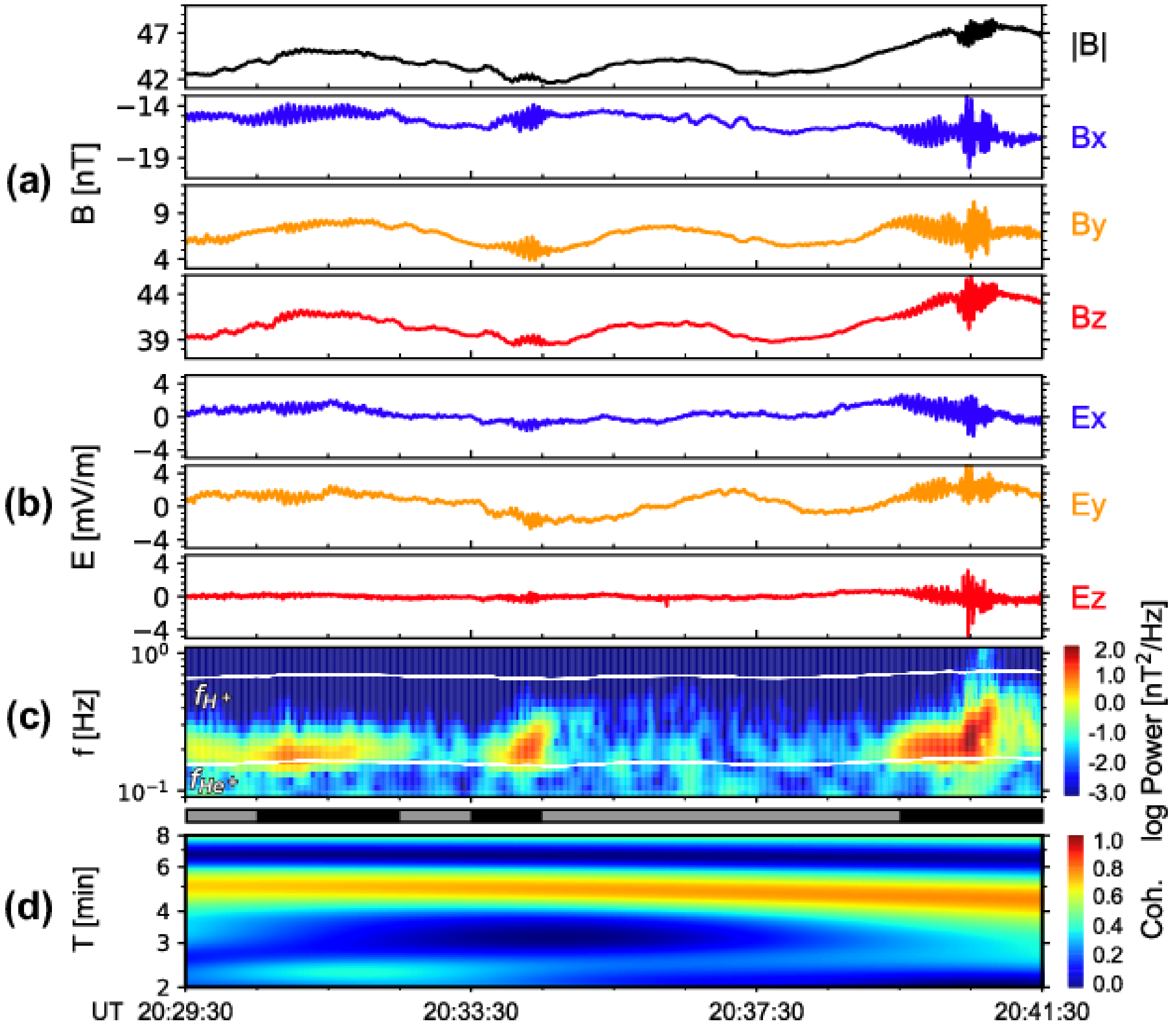

Supplementary Fig. 3. Magnetic and electric field observations in the January 7, 2019, event. (**a**) The magnitude and three GSE components of the magnetic fields. (**b**) The three GSE components of the electric fields. (**c**) Dynamic spectra of the magnetic field derived from the Fourier transform. The two white curves represent the gyro-frequency of H^+ and He^+ ions. The bar below this panel marks the time periods when EMIC waves are present (black) and absent (grey). (**d**) The cross-wavelet correlation coefficient between the ULF wave Ey (panel b) and the EMIC wave power at 0.22 Hz (panel c).

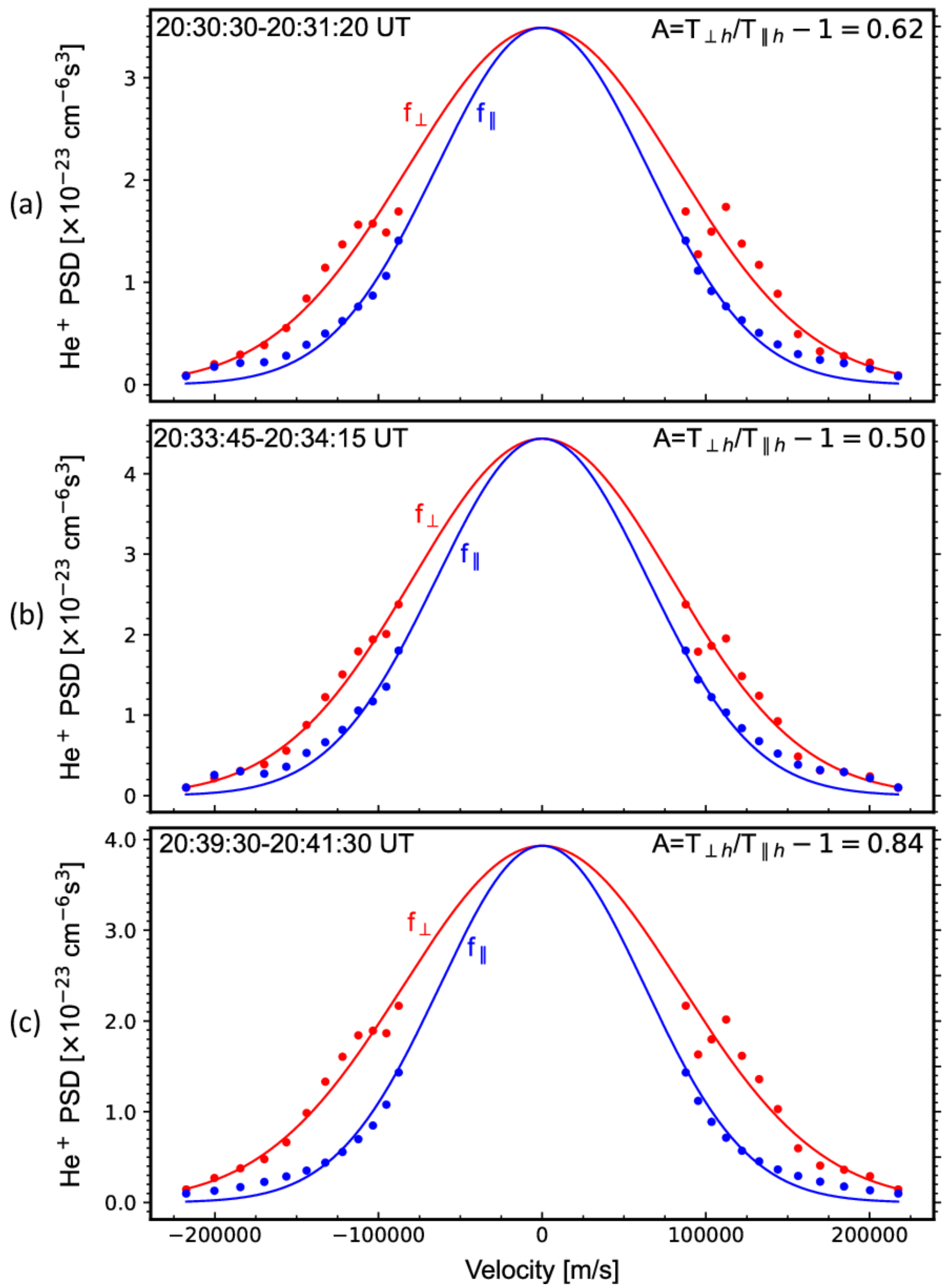

Supplementary Fig. 4. The PSDs and temperature anisotropy of He^{+} ions in the January 7, 2019, event. HPCA fast survey mode data is used here. (**a**), (**b**) and (**c**) correspond to the three EMIC-wave packets. In each panel, the blue and red dots represent the PSDs for field-aligned motion (0°-30° and 150°-180° PA) and perpendicular motion (75°-105° PA), respectively, with the corresponding curves showing the fit of bi-Maxwellian distributions.

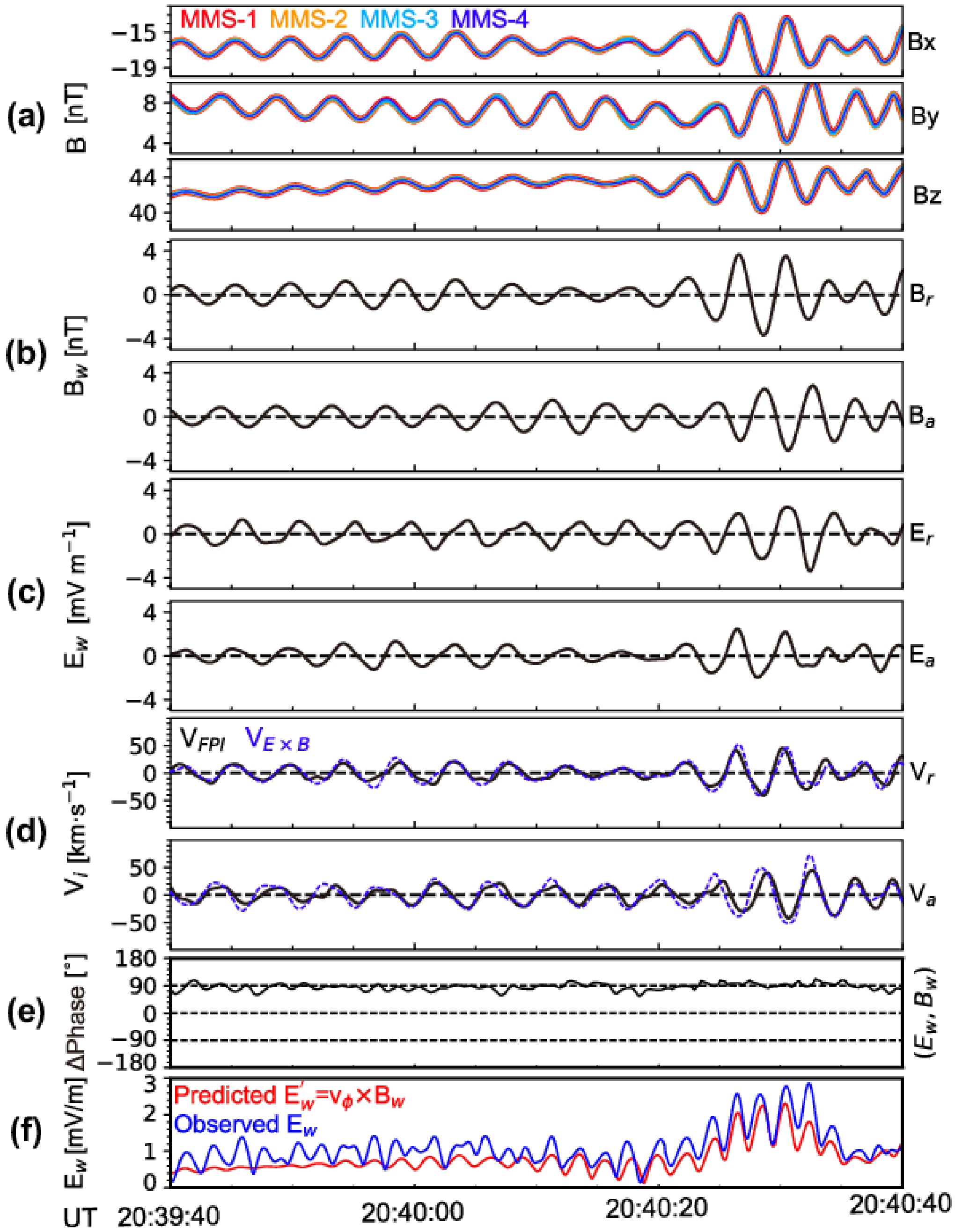

Supplementary Fig. 5. EMIC-wave magnetic and electric fields in the January 7, 2019, event. (**a**) Three GSE components of the magnetic fields. (**b** and **c**) EMIC wave perpendicular magnetic and electric fields in the FAC, respectively. (**d**) EMIC wave-induced ion bulk motion. The black and blue curves represent the FPI measurements and the E×B drift velocity calculated from the field measurements, respectively. (**e**) The phase differences between the observed EMIC magnetic fields and electric fields. +90° indicates that the waves are left-hand circularly polarized and anti-parallel propagating. (**f**) The amplitude of the observed (blue) and predicted (red) electric fields. The agreement between the observations and the predictions indicates that the electric field instruments perform well in this event.

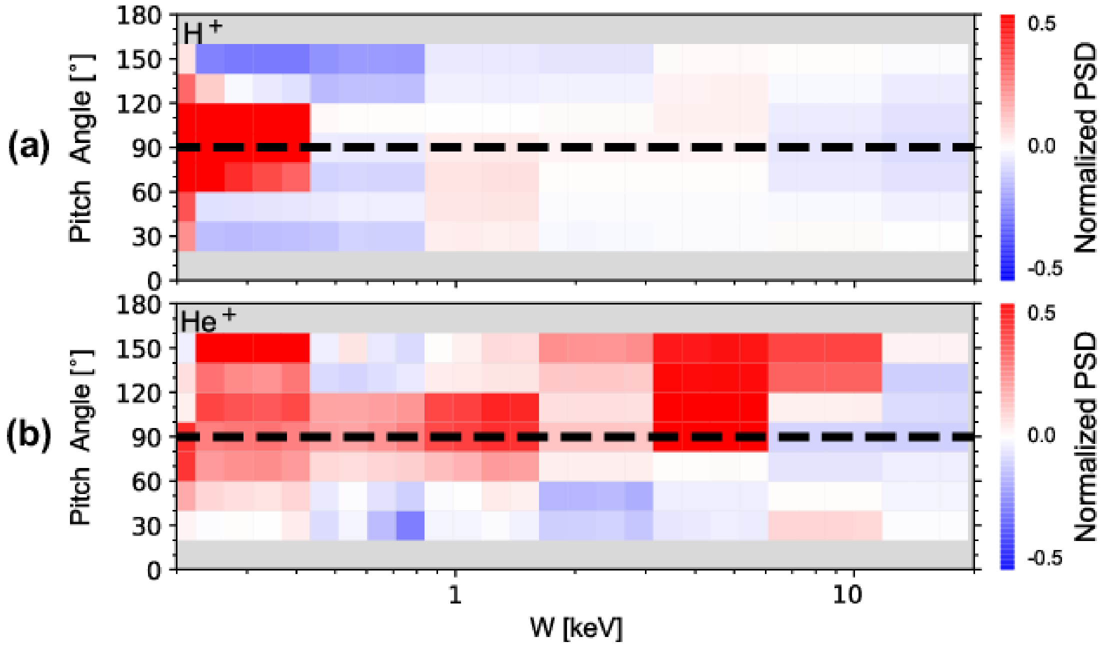

Supplementary Fig. 6. Ion composition observations obtained by HPCA in the January 7, 2019, event. The fast survey mode data is used here. (**a**) H^+ ion residual PSDs, defined in the same manner as Fig. 3a. (**b**) He^+ residual PSDs.

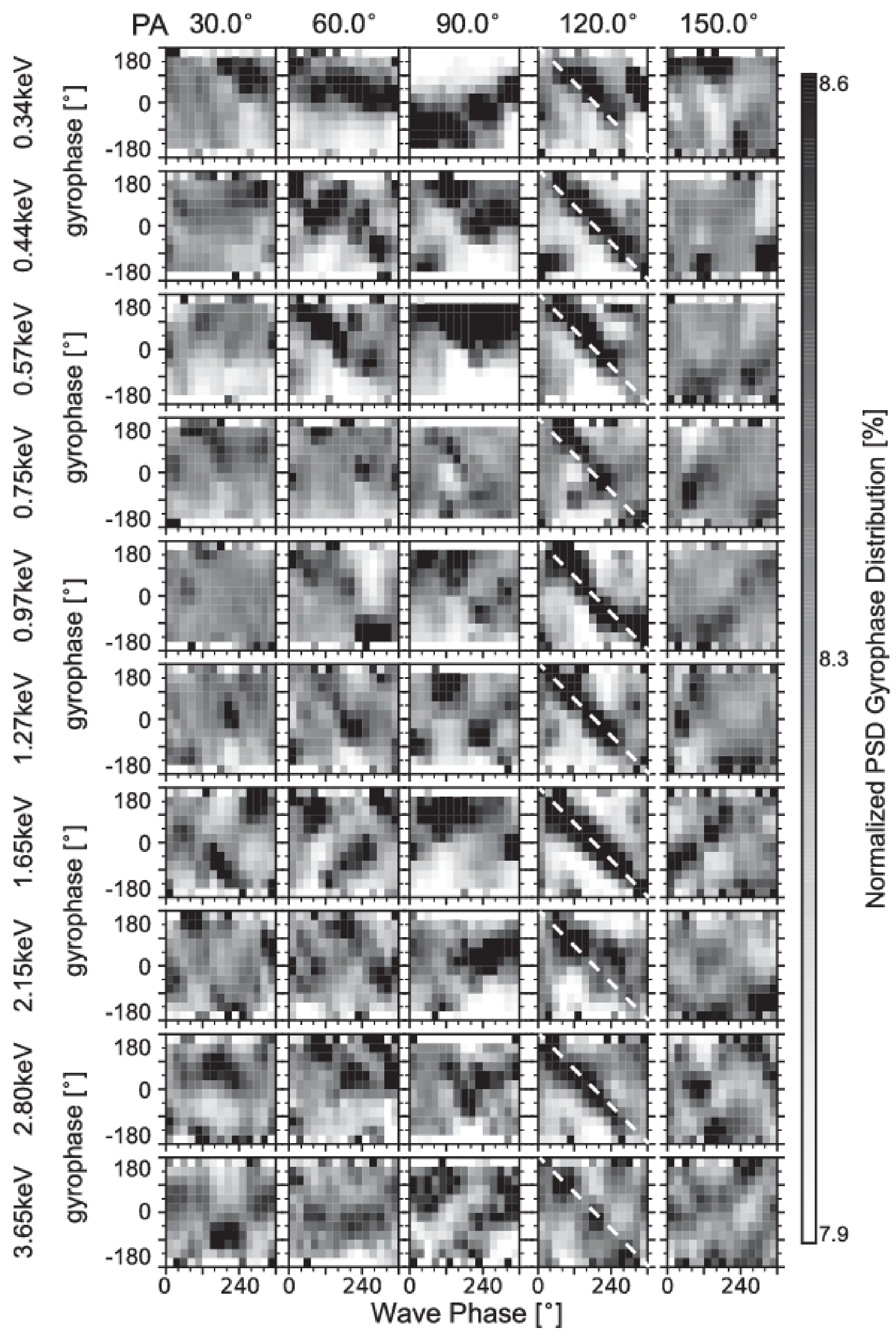

Supplementary Fig. 7. Gyrophase bunching observations in the January 7, 2019, event. Each panel corresponds to an energy and a PA. The color codes represent the normalized PSDs derived from a superposed epoch analysis on the five wave cycles during 20:40:07-20:40:32 UT. The white lines mark the gyrophase of $-\mathbf{B}_{\mathbf{w,EMIC}}$. FPI burst mode data is used here.

Supplementary Methods

Treatment of the EMIC wave magnetic and electric fields. Magnetic fields and electric fields were measured by the Fluxgate Magnetometers (FGM) with a sampling rate of 16 Hz and the Electric Field Double Probe (EDP) with a sampling rate of 32 Hz, respectively. The original data is given in the GSE coordinate system. To separate EMIC wave fields from the background, we first performed a windowed fast Fourier transform to determine their center frequency. Then, a band-pass filter was applied to obtain the EMIC wave fields (the passband is 0.05-0.8 Hz in the second event). Finally, the obtained EMIC wave fields were transformed into the DBCS or the FAC system, when necessary.

A singular value decomposition (SVD) analysis[1] was applied to determine the properties of the EMIC waves. Both in the first and second event, when wave power is large, the SVD analysis shows that the degree of polarization and ellipticity are close to 1 and -1, respectively, indicating the EMIC waves are left-hand circularly polarized. Also, the SVD analysis reveals that the wavevector of these waves is mostly field-aligned. All of these properties are consistent with the expectation for EMIC waves. We further derived the angle between the wave Poynting flux and the background magnetic fields in the second event. The results suggest that the EMIC waves propagate anti-parallel to the background magnetic fields, as the obtained angle is ~180°. It is noted that, in both the two events, the propagation and polarization properties are consistent through all the EMIC wave packets.

In the second event, the observed electric fields are used to calculate the energy exchange between EMIC waves and ions. To qualify the performance of the electric field instruments, we checked the phase difference between the magnetic and electric fields of the EMIC waves. As shown in Supplementary Fig. 5e, the phase difference is approximately +90° in the whole time interval of interest, agreeing with the expectation. We also checked the amplitude of the electric fields. The blue and red curves in Supplementary Fig. 5f respectively give the amplitude of the observed wave electric fields and the one derived from $\mathbf{E'}_{\mathbf{w},\mathbf{EMIC}} = \mathbf{v}_{\boldsymbol{\phi},\mathbf{EMIC}} \times \mathbf{B}_{\mathbf{w},\mathbf{EMIC}}$, where $\mathbf{E'}_{\mathbf{w},\mathbf{EMIC}}$, $\mathbf{B}_{\mathbf{w},\mathbf{EMIC}}$ and $\mathbf{v}_{\boldsymbol{\phi},\mathbf{EMIC}}$ denotes the predicated wave electric fields, the observed wave magnetic fields, and the phase velocity of the waves (see below). The predicted amplitude roughly matches the observed amplitude. The absolute and relative differences are ~0.3 mV/m and ~25%, respectively. Therefore, we conclude that the electric field instruments performed well in the second event, ensuring a reliable calculation of the energy transfer between ions and wave fields.

Treatment of ion measurements. Because of its special design, the FPI (Fast Plasma Investigation) instruments[2] in burst mode are capable of providing 4π ion measurements with a sampling time (150 milliseconds) much smaller than the spacecraft spin-period (20 seconds). Such a small sampling time provides an opportunity to investigate the response of ions to EMIC waves and the energy transfer in the interactions. Besides, fast mode FPI data, whose sampling time is ~4.5 seconds, is also used in this paper, to reveal the response of ions to ULF waves. The FPI instruments measure ions from ~2.2 eV to 20 keV. This energy range is divided into 32 channels. The original FPI data is given in the DBCS, with an angle-resolution of $11.25°$. To construct pitch angle distributions and gyrophase distributions, we first transformed original data into the FAC system. Then, a spherical coordinate system is defined according to the FAC system, with the main axis aligned with the FAC z-axis. According to the definition of the FAC system, the polar angles and azimuthal angles of this spherical coordinate system are particles' pitch angles (α) and gyrophase angles (ϕ), respectively. Pitch angles are then divided into 5 intervals: $15° - 45°$, $45° - 75°$, $75° - 105°$, $105° - 135°$, and $135° - 165°$. Gyrophase angles, which range from $0°$ to $360°$, are evenly divided into 12 intervals. In this way, we obtained a $5\alpha \times 12\phi$ grid. Finally, ion measurements in the FAC system were binned according to the $\alpha - \phi$ grid, giving corresponding pitch angle distributions and gyrophase distributions. The angle-resolution of the final data is $30°$. Angle resolution is reduced, in order to get enough counts in one bin. After integration over time, angle and the four MMS spacecraft, standard errors are generally one order of magnitude less than the measurements.

To further depress noise, superposed epoch analysis is applied (Fig. 4a and Supplementary Fig. 7). First, we isolated five successive wave cycles from 20:40:07-20:40:32 UT. Each wave cycle starts at the time when $\mathbf{B}_{\mathbf{w,EMIC}}$ points towards the opposite direction of the FAC radial axis, and ends at the time when $\mathbf{B}_{\mathbf{w,EMIC}}$ returns to this direction again. The lengths of the five wave cycles are about 4.5 s, the period of the EMIC waves. Then, we linearly interpolated ion gyrophase distributions in the five wave cycles to make a uniformly spaced time series with 4.5-s duration and 300-ms time resolution. Next, the five gyrophase distributions are superposed with respect to time. For convenience, the results of the superposition are shown as a function of wave phase ranging from $0°$ to $360°$. Finally, the resulting distributions are normalized to make that, at any given wave phase, the sum of the normalized PSDs is a unit.

The FPI instruments do not distinguish ion species. Ions of different species but with the same energy/charge ratio will be regarded as the same ions by them. To get the information on ion species, here we used data obtained by the HPCA (Hot Plasma Composition Analyzer) instruments[3], which can resolve ion species (H^+, He^+, He^{++}, O^+) with a time-of-flight technique.

In this event, the counts of HPCA burst mode data are too low to provide reliable measurements. Thus, only fast survey mode data is used in this paper. In this mode, the HPCA instruments provide a 4π measurement of ions from ~1.4 eV/q to 37 keV/q every 10 seconds (i.e., the 1/2 spin of the spacecraft). Taking into account the data decimation, the effective angular resolution of the data is about 45°, and the effective number of the energy channels is 16. The HPCA data is processed in a way similar to the FPI data. For H^+ and He^+ ions, the HPCA PSD measurements are generally three orders of magnitude larger than the one-count level. In addition, after integration over time, angle and spacecraft, statistical errors in the HPCA data (shown as error bars in Fig. 3b) are one order of magnitude less than the measurements. All these suggest the HPCA observations of H^+ and He^+ ions are reliable. Also, we note that the wave-related signals (Figs. 2c) observed in He^+ ion data cannot be attributed to H^+ ion contamination, since no corresponding signal is observed in H^+ ion data.

In the second event, the PSD enhancements for 200-600 eV He^+ ions are suggested to be caused by drift-bounce interactions rather than ULF wave-induced $E \times B$ drift. This suggestion is supported by estimating the contributions to the PSD enhancements from the high-energy tail of the background warm He^+ ions. For example, during 20:39:40-20:40:40 UT, the energy spectrum of He^+ ions is composed of two components: a power-law distribution below ~100 eV corresponding to the background warm He^+ ions and a bump-on-tail distribution around 200-600 eV corresponding to the PSD enhancements. By fitting the power-law distribution and extending the resulting curve to 200-600 eV, the contributions from the background warm component to the PSD enhancements are estimated to be less than 20%. Therefore, the high-energy tail of the background warm He^+ ions does not contribute much to the PSD enhancements at 200-600 eV.

Supplementary References

1. O. Santolík, M. Parrot, F. Lefeuvre, Singular value decomposition methods for wave propagation analysis. *Radio Sci*. **38**, 1 (2003).
2. C. Pollock, T. Moore, A. Jacques, J. Burch, U. Gliese, Y. Saito, T. Omoto, L. Avanov, A. Barrie, V. Coffey, J. Dorelli, D. Gershman, B. Giles, T. Rosnack, C. Salo, S. Yokota, M. Adrian, C. Aoustin, C. Auletti, S. Aung, V. Bigio, N. Cao, M. Chandler, D. Chornay, K. Christian, G. Clark, G. Collinson, T. Corris, A. De Los Santos, R. Devlin, T. Diaz, T. Dickerson, C. Dickson, A. Diekmann, F. Diggs, C. Duncan, A. Figueroa-Vinas, C. Firman,

M. Freeman, N. Galassi, K. Garcia, G. Goodhart, D. Guererro, J. Hageman, J. Hanley, E. Hemminger, M. Holland, M. Hutchins, T. James, W. Jones, S. Kreisler, J. Kujawski, V. Lavu, J. Lobell, E. LeCompte, A. Lukemire, E. MacDonald, A. Mariano, T. Mukai, K. Narayanan, Q. Nguyan, M. Onizuka, W. Paterson, S. Persyn, B. Piepgrass, F. Cheney, A. Rager, T. Raghuram, A. Ramil, L. Reichenthal, H. Rodriguez, J. Rouzaud, A. Rucker, Y. Saito, M. Samara, J.-A. Sauvaud, D. Schuster, M. Shappirio, K. Shelton, D. Sher, D. Smith, K. Smith, S. Smith, D. Steinfeld, R. Szymkiewicz, K. Tanimoto, J. Taylor, C. Tucker, K. Tull, A. Uhl, J. Vloet, P. Walpole, S. Weidner, D. White, G. Winkert, P.-S. Yeh, M. Zeuch, Fast Plasma Investigation for Magnetospheric Multiscale. *Space Sci. Rev.* **199**, 331–406 (2016).

3. D. T. Young, J. K. Burch, R. G. Gomez, A. De Los Santos, G. P. Miller, P. Wilson IV, N. Paschalidis, S. A. Fuselier, K. Pickens, E. Hertzberg, C. J. Pollock, J. Scherrer, P. B. Wood, E. T. Donald, D. Aaron, J. Furman, D. George, R. S. Gurnee, R. S. Hourani, A. Jacques, T. Johnson, T. Orr, K. S. Pan, S. Persyn, S. Pope, J. Roberts, M. R. Stokes, K. J. Trattner, J. M. Webster, Hot plasma composition analyzer for the magnetospheric multiscale mission. *Space Sci. Rev.* **199**, 407-470 (2016).
